# Is the mainstream construction of mood disorders resistant to systemic thinking?

**DOI:** 10.3389/fpsyt.2023.1270027

**Published:** 2024-01-19

**Authors:** Lisa C. Fellin, Ekaterina Zizevskaia, Laura Galbusera

**Affiliations:** ^1^Department of Human and Social Sciences, University of Bergamo, Bergamo, Italy; ^2^Department of Psychiatry and Psychotherapy, Brandenburg Medical School, Brandenburg, Germany

**Keywords:** causal explanations, systemic explanations, psychopathology, etiology, mood disorders, major depression disorder (MDD), relational, biomedical

## Abstract

**Introduction:**

In this study we explore how the diagnostic category of mood disorders is constructed in two handbooks of Psychopathology as an example of the mainstream construction of psychopathology. Despite the increasing criticism and lack of evidence, the debunked chemical imbalance theory of the etiology of depression still dominates the professional and pop/folk understanding and interventions.

**Methods:**

We analysed the breadth of the inference field and the type of etiopathogenetic contents of the explanations of mood disorders using the “1to3” Coding System.

**Results:**

Our findings show that the dominant explanations draw almost exclusively onto monadic explanations, followed by limited dyadic ones. Intrapersonal etiopathogenetic contents prevailed, and biomedical explanations were dominant in both textbooks.

**Discussion:**

We critically discuss the underpinnings of these results and address the clinical implications of these biased representations, as well as potential alternative approaches to psychopathology.

## Introduction

### Beyond the bio-bio-bio: from “neurochemical to inter-dependent selves”

Stemming from a critical and socio-constructionist epistemology, this paper critically explores the current dominant constructions of psychopathology through the analysis of the definitions of mood disorders and their etiological explanations, which are put forward in a selection of mainstream psychiatric textbooks. We conclude by pointing towards an alternative approach.

Founded as a discipline by Karl Jaspers, psychopathology is the scientific study of mental disorders, which should envisage not only the nosological description or categorization of symptoms and syndromes, but also the development of *meaning-ful* etiopathogenetic accounts.

Over the last 2 centuries though, it has progressively lost its original mission and interest for meaning (re-)making, hermeneutics and phenomenology, to confine itself within the absolute domain of nosographic psychiatry in the attempt to secure itself the same reputation of other branches of medicine. Mesmerised by the hyper-biologistic assumptions that were bringing high expectations and then largely debunked, psychiatry ended up bracketing all the other possible explanations and clinical theories and ultimately, therapeutic approaches. This bio-medical model aiming solely at nosographic classification and at postulating pathophysiological causes of mental disorders put forward explanatory models based on monadic inference, determinism and linear causality and, ultimately, to hermeneutic closure (1).

Nevertheless, during the last century, different critical psychiatry and therapeutic approaches, and especially systemic theories, attempted to resist this soaring trend and breached the shallow limits of nosographic psychiatry, bringing back psychopathology to its very core: emotions and meaning [e.g., (2–4)]. These attempts go beyond the *theoretical* recognition of the role of relational, social and contextual aspects in the development of mental disorders, thus extending etiopathological models beyond the monad; however, these factors are still largely neglected in mainstream clinical practice and thinking.

Moreover, psychopathology and related terms (e.g., *psychiatric disorder, emotional distress,* and *mental illness*) are often used as interchangeable, disregarding the very different ontological and epistemological underpinnings of these constructs.

Within our constructionist, complex and critical perspective, we consider psychopathology not as an “objective” scientific term, but rather as a cultural, political and social *construction* (5–7), largely or entirely determined by different ontologies and epistemologies, as well as societal, cultural and professional values, biases and allegiances. As maintained by McNamee and Gergen (8): “The mental health profession is not politically, morally, or valuationally neutral. Their practices typically operate to sustain certain values, political arrangements, and hierarchies of privilege” (p. 2).

Addressing psychopathology from this perspective is pivotal, so that what is categorised as “disorder” can be understood and located as by-product of particular historical, professional and socio-cultural constructions tied to financial and lobby interests, rather than a reification, i.e., acritically assumed as objective descriptions of universal and stable categories of human experience stripped of their contextual determinants.

Within this epistemological framework, all phenomena are socially constructed and are therefore also contingent and open to change (deconstruction) rather than being static and chronic. For instance, the meaning of “depression” is always determined by the socio-political, historical, and cultural context within which it is constituted, as well as by its relational dynamics (9–11). This motivates us to study both collective and individual processes of meaning-making in mental health, including the corresponding academic, mediatic and industry discourses within which these processes take place.

As Galbusera and Fellin (12) previously argued, the dominant third-person approach (TPA) of mainstream psychopathology research and practice is mostly based on descriptive diagnostic categorizations such as those regulated by the Diagnostic and Statistical Manual of Mental Disorders (13). As well known, since its third edition the DSM claims a position of a-theoreticity, but it actually relies on an epistemology of logical empiricism and on physicalist ontology. Therefore mainstream research and practice in psychopathology reflect the very same bias. The TPA constructs symptoms in a “game of semantics” that Timimi (14) exposes like this:

Symptoms become much more than descriptive constructions: they are reified providing the illusion that the disorder itself exists as a natural object. This easily leads to etiological theories that link mental distress to supposed biochemical or genetic causes (and therefore, mostly pharmaceutical interventions). In this approach to diagnosis, individuals are equated to their diagnostic label and therefore stigmatized or even alienated and dehumanized. (p. 116)

The awareness that individuals are always to be conceived within the complex systems in which they are embedded originally stems from the clinical field of systemic therapy. From this interdisciplinary perspective it is nowadays broadly recognized that the human psyche—and thus its *dis*-orders—cannot be conceived as being disconnected from its body and from the systemic intertwined loops within its environment. Although mainstream psychopathology is in principle based on bio-psycho-social assumptions, the psycho-*social* aspects are actually neglected when it comes to conceptualising and treating “mental” disorders (15).

Indeed, also in response to the strong opposition of critical psychiatry in 1950–1960, the introduction of the bio-psycho-social approach in the ‘70s was supposed to bring forward the complex interplay of social, biological and psychological factors. Yet this has remained only a theoretical basis for understanding and explaining mental disorders, not reflected in mainstream clinical practice and training, that progressively collapsed into solely drug prescriptions consistent with what Schultz labels *neuroessentialism* (16). Back in 2005, Dr. Steven Sharfstein (17), then President of the American Psychiatric Association, already admitted that:

“If we are seen as mere pill pushers and employees of the pharmaceutical industry, our credibility as a profession is compromised. As we address these Big Pharma issues, we must examine the fact that as a profession, we have allowed the bio-psycho-social model to become the bio-bio-bio model.”

### Constructions and de-constructions of psychopathology

For decades, the dominant “biomedical model” approach has been promoted by various forces, the strongest being insurance companies, biological psychiatry and its financial ties with drug companies (18, 19). Amongst its numerous critics (1, 9, 20–28), there are now several prominent APA psychiatrists and former heads of DSM task forces (29).

Even the former Director of the U.S. National Institute of Mental Health (NIMH), Insel (30) in *Healing: Our Path from Mental Illness to Mental Health* blatantly acknowledged that compelling research forced psychiatry to discard the “chemical imbalance theory” of mental illness. But Insel has swiftly jumped on the new psychiatry’s bandwagon: the “circuitry defect” theory of mental illness. In this ultimate attempt to absolve psychiatry, Pies (31) even tried to dismiss the ‘chemical imbalance’ as an “urban legend” of mental illness (32–34); indeed the Emperor’s new clothes is the new (unsupported) rebranding of mental illnesses as ‘connectional’ or ‘brain circuit disorders’.

According to critics like Levine (35) and Gøtzsche (36), Insel (30) has made crystal clear: (1) psychiatry’s worsening treatment outcomes; (2) psychiatry’s jettisoning of its chemical imbalance theory of mental illness; and (3) the scientific invalidity of the DSM (“The DSM had created a common language, but much of that language has not been validated by science”). The DSM has been widely criticized for various reasons, not least because the number of official mental disorders recognized by the American Psychiatric Association has increased from six in the mid-19th century to close to 300 in the DSM-5 (37). Between 1952 and 2013, the number of pages in the DSM increased from 130 (mostly appendices) to over 900. At the same time, as abovementioned, nosographic manuals have progressively obliterated any explicit aetiological hypotheses or factors from their criteria. Current manuals claim to hold an atheoretical and “agnostic” approach to etiology and to maintain a descriptive approach to classification of mental disorders. But all the above-mentioned authors have highlighted how this is misleading and how current diagnostic categorizations indeed imply a bio-medical etiology, starting from the very definition of disorders.

However, neither Insel nor Pies acknowledge the underlying inconvenient truth, i.e., the possibility that the bio-psychiatry’s medical model that constructs human beings as “bio-chemically-electrically defective in need of bio-chemical-electrical treatments is a failed paradigm” (36).

Nevertheless, the momentum for a fundamental shift in mental health that many critical authors have advocated for long might, finally, approach, also thanks to very high profile endorsements. Dr. Pūras, UN Special Rapporteur (38) and Lithuanian psychiatrist, called for a drastic move away from drug-company-supported biological explanations of *human distress*. In 2019, Pūras argued:

“Current mental health policies have been affected to a large extent by the asymmetry of power and biases because of the dominance of the biomedical model and biomedical interventions. This model has led not only to the overuse of coercion in case of psychosocial, intellectual and cognitive disabilities, but also to the medicalization of normal reactions to life’s many pressures, including moderate forms of social anxiety, sadness, shyness, truancy and antisocial behaviour.”

The World Health Organization (39) echoed this call in a paper entitled “Guidance on Community Mental Health Services: Promoting Person-Centred and Rights-Based Approaches,” critiquing the overly biological approach in mental health and calling for fundamental changes, or even a revolution. Both these groundbreaking reports pivoted the etiological emphasis to the social determinants and structural inequalities of mental health, such as violence, discrimination, poverty, exclusion, isolation, and unemployment.

The WHO’s report (40) states:

"The predominant focus of care in many contexts continues to be on diagnosis, medication and symptom reduction. Critical social determinants that impact on people’s mental health such as violence, discrimination, poverty, exclusion, isolation, job insecurity or unemployment, lack of access to housing, social safety nets, and health services, are often overlooked or excluded from mental health concepts and practice. This leads to an over-diagnosis of human distress and over-reliance on psychotropic drugs to the detriment of psychosocial interventions.”

"A fundamental shift within the mental health field is required, in order to end this current situation. This means rethinking policies, laws, systems, services and practices across the different sectors which negatively affect people with mental health conditions and psychosocial disabilities, ensuring that human rights underpin all actions in the field of mental health. In the mental health service context specifically, this means a move towards more balanced, person-centered, holistic, and recovery-oriented practices that consider people in the context of their whole lives, respecting their will and preferences in treatment, implementing alternatives to coercion, and promoting people’s right to participation and community inclusion."

Beeker et al. (20) put forward a two-way conceptual model of this growing psychiatrization of society, distinguishing between top-down and bottom-up agents and processes. Among the top-down forces we would encompass also the curricula training of new mental health professional, in particular that of physicians, as psychiatrists and GPs are the main “pill pushers” and the strongest direct influencers of patients’ biological explanations of their distress, together with direct-to-consumer (“DTC”) pharmaceutical advertising (1, 41). Beeker et al. (20) also call for interdisciplinary research investigating causes, mechanisms, and effects of psychiatrization, to which we aim to contribute with this paper.

Several critical studies have explored how different versions of mental health are constructed in different contexts and the effects of various variables on these (9, 42, 43). It is important to keep in mind that although dominant stereotypes about mental health exert a powerful role (e.g., when defining what it means to be healthy or ill), different individuals and groups, in various contexts, may construct a variety of different disorders in constantly changing feedback loops (1, 20).

Another contribution comes from Davis’ (1) interview study which found that doctors were the primary source of influence that make lay people keener to embrace the biological explanations of emotional distress, disregarding other factors that were previously considered more relevant, and which orient them towards more pharmacological solutions rather than psychotherapy, consistently with Watson and Beshai’s findings (44). Citing an older US study by Jones, Kahn, and Macdonald (45), Davis (1) illustrates also how these lay people attributions have shifted from 1970s onwards: patients shifted from relational explanations towards bio-glitch and quick fix attitudes. These findings are also consistent with other research targeting populations not directly influenced by physicians or DTC ads (46, 47). O’Neill, Stapley, Stock, Merrick and Humphrey’s (48) study found that UK adolescents resorted mostly to interpersonal and contextual factors to explain their emotional distress.

Ugazio and colleagues (49) found that clients in private (systemic) psychotherapy with psychologists resorted mostly to interpersonal explanations rather than biomedical ones. Other research conducted with Italian lay populations (50, 51) found that mental disorders (including depression) are mostly seen as a reaction to significant current life events and psycho-social stressors and that should be overcome with the help of health professionals (mainly psychologists) and/or support of significant others. This is consistent with findings from other countries [see (50)]. Magliano et al. (46) also explored how Italian psychology students’ constructions of 2 disorders changed during their 5-year academic training. Interestingly, first year students more frequently mentioned psychosocial factors among the causes of depression and believed more in the usefulness of psychotherapy and less about drugs; they also had more prognostic optimism; at the end of their education, students cited heredity as a cause more than at first year. This finding confirms the increasing relevance assigned to biogenetic factors at disadvantage of a more balanced biopsychosocial model and therapeutic approach to this disorder.

We can hence argue that current curricula taught to mental health professionals reflect these mainstream theoretical underpinnings, and hence educate future mental health professionals to practice according to this dominant construction of disorders, especially for those diagnostic categories associated with important incomes for “Big Pharma” and growing sectors of possible life-long consumers (6, 7, 52). In the last decades, psychiatrists almost stopped undertaking psychotherapy training (53) and Tadmon and Olfson (54) highlight that more than half of psychiatrists in a US nationally representative survey claimed they do not provide psychotherapy of any kind. Moreover, the percentage of psychiatrist visits involving psychotherapy dropped more than 50% between 1996 and 2016 and patients from minority groups and/or facing socioeconomic disadvantages have the lowest likelihood of receiving psychotherapy from their psychiatrists.

Moran (55), a founding member of the APA Caucus on Psychotherapy, pointed out that the disparity revealed by Tadmon and Olfson (54) uncovers a wider public health crisis, mostly driven by an insurance industry that disincentivizes treatment aimed at recovery by the most highly trained practitioners and instead has been focused on “mere crisis stabilization.” He noted that this includes psychotherapy in general and its provision by psychiatrists, now in a “professional identity crisis.”

The growing research field of critical mental health studies is hence also relevant when it comes to understanding assumptions among mental health professionals and how these inform and shape lay people’s and patients’ ones. We shall take the paradigmatic example of mood disorder to illustrate this dominating and problematic trend.

### De-constructing mood disorders

According to the World Health Organization (56), depression is a common mental disorder. Globally, more than 280 million people of all ages suffer from, or better, are *diagnosed* with depression (56).

Despite depression being known since ancient times, up to 40 years ago it was a rare psychopathology; however, the incidence of depression globally rose from 172 million in 1990 to 258 million in 2017, indicating a growth of 49.86% (57). Despite this trend can reflect the growth of the population (58), some authors question whether this is a case of disease mongering (59) and over diagnosis.

Depression is also a leading cause of disability worldwide and is a major contributor to the overall global burden of disease. While the burden on health-care systems and societies is allegedly still underestimated and projected to grow constantly, the current bio-psychiatrization is also adding to this economic burden (20).

The idea that depression is caused by a brain chemical imbalance (i.e., lowered serotonin) has been described at the same time as the “dominant cultural story of depression etiology” [(33), p. 411] and as an “urban legend” (31, 60). Beyond this lack of evidence, the WHO website warns that:

Barriers to effective care include a lack of resources, lack of trained health-care providers and social stigma associated with mental disorders. Another barrier to effective care is inaccurate assessment. In countries of all income levels, people who are depressed are often not correctly diagnosed, and others who do not have the disorder are too often misdiagnosed and prescribed antidepressants.

More recently, the World Health Organization’s (61) Mental Health Gap Action Programme (mhGAP) guideline for mental, neurological and substance use disorders has established that antidepressant (including SSRI) are not the first line treatment for depression and should only be prescribed when psychological interventions are not available and put an emphasis on assessment of psycho-social stressors.

However, the dominant narrative is still entrenched in professional, mediatic and pop discourses, as it has altered the way people think about their moods in terms of brain chemicals and, by extension, their very concept of themselves as ‘neurochemical selves,’ with profound implications for their sense of agency and self-efficacy (62), as well as possibility of recovery and the relevance of socio-relational factors and possible aids.

As already mentioned, several leading psychiatrists have been claiming that the chemical imbalance theory is an ‘urban legend’ that was never ‘seriously propounded by well-informed psychiatrists’ as the aetiological cause of depression (31, 60).

In this paper, we explore how this theory is still influential through academic textbooks, although officially conveniently dismissed as an “urban legend” for well over a decade now.

Ang et al. (32) have analysed different sources of the serotonin theory of depression to challenge Pies’ defense of the ‘urban legend’. Their exploration of the status quo in the scientific literature includes also psychiatry and psychopharmacology text-books published between 1990 and 2012 in the United Kingdom and United States. All the textbooks reviewed by the Authors acknowledged that the serotonin hypothesis is not necessarily proven, and some stressed the provisional nature of research findings on the biological basis of depression. All the textbooks dedicated substantial space to the discussion of serotoninergic factors and ultimately supported the theory. However, Ang et al. (32) did not analyse in depth the different types of explanations provided in these books nor how much emphasis is given to the psycho-social and systemic-relational factors. Indeed, handbooks are very influential for mental health professionals in training as they are considered an established and prestigious source of references that informs their education and future work. They are regarded as the synthesis of the up-to-date evidence base and state of the art in the field and most likely they will also direct future training orientations for professionals and treatment indications for clients.

The breadth of the inference field in mainstream psychopathological accounts has not been an object of scientific inquiry yet. With this study we seek to assess the extent to which mainstream psychopathology reflects the current scientific standard of embodied, extended, enacted and embedded approaches to the human mind, which are based on systemic and complex thinking (63, 64).

## Materials and methods

Our study aims at exploring this overarching question: are handbooks of psychopathology up to date with a systemic and relational epistemology?

Or are they still imbued with the predominant bio-bio-bio model?

To answer these questions, this study analyses the breadth of the inference field of psychological symptoms explanations in two mainstream psychopathology manuals. We take the DSM-5, one of the two main diagnostic classification systems, as representing the current mainstream approach. Yet, since the classification system is *per se* conceived as being merely descriptive, we focus on two psychopathology manuals, reflecting the DSM nosology with etiopathogenetic accounts.

We took into consideration as a case study one of the most controversial diagnostic categories of psychopathology (33, 63): the mood disorders, which are at the very core of the heated debate discussed above.

The main research questions for this study are of exploratory nature:

1. What breadth of the inference field prevails in psychopathological accounts of mood disorders in these textbooks?
2. What kind of etiological contents (factors/causes) feature in psychopathological accounts of mood disorders in these textbooks?

To answer these questions, this study considered all the explanations related to mood disorders put forward in two mainstream psychopathology academic manuals (64, 67). In this sense, each text can be studied in relation to both the professional discursive practice, in which the texts are produced and/or consumed, and the surrounding wider sociocultural context, which also contributes to the meaning of any given text.

Both texts are professional manuals on psychopathology that claim to offer guidelines for students, educators, and practitioners. They were chosen for analysis as texts that can be perceived as trustworthy, given their publication by leading publishers such as Guilford Press and the American Psychological Association. Furthermore, both texts are recommended on the APA website, a leading and influential institution worldwide.

### Content analysis

As applied in this study, we see each articulation about mood disorders and related interventions as text, and the academic psychiatric culture as the discursive practice—in other words, the immediate context within which these meaning-making processes take place. It is more difficult to account for the wider sociocultural practice or political context, because it potentially encompasses “everything” and would require a different research approach. In this analysis, however, we have focused on a limited number of categories that prove relevant with regards to defining what it means to be “depressed,” why and how a person diagnosed with depression should get treated.

We focused on the three following categories of explanations:

1. Symptom explanations

Example: “Together these studies provide strong evidence that rumination in the context of either naturally occurring or experimentally induced depressed mood maintains dysphoria, enhances negative thinking, and impairs problem solving” [(67), p. 34].

1. Therapeutic change explanations

Example: “Patients with bipolar forms of mood disorder can respond well to the antidepressant medications, especially when they are in the depressed phase of the disorder” [(64), p. 42].

1. Etiology explanations

Example: “Neurotic depressions belonged to a larger category of nervous disorders, which were thought to stem from abnormalities of the nerves, fibers, and organs” ((64), p. 31).

We analysed the breadth of the inference field of the explanations using the “1 to 3” Coding System (68). This coding system allows distinguishing between monadic, dyadic (unidirectional or bidirectional) and triadic (triadic or systemic) explanatory models. This has been complemented by the additional categories included by Ugazio et al. (49) to analyse the type of contents/causes of the explanations [amended from Schweizer et al. (69)].

We put forward the following hypotheses: that both texts utilize more monadic and dyadic explanations than triadic explanations (H1); and that intrapersonal (H2) and biomedical explanations (H3) prevail in both texts.

### Coding procedure

The coding and classifying system “1 to 3: from the monad to the triad” (68) was applied to the chapters.

The detected explanations are classified according to the inference field, using five categories, and operationalized as follows. The examples provided come from the present data corpus.

1. *Monadic.* The explanation is sought within the individual.
  Example: “Indeed, neuroticism has been found in several studies to predict the development of depressive episodes” [(64), p. 26].
2. *Unidirectional dyadic.* The explanation involves two characters, only one of which has an active influence on the other.
  Example: “Depression is viewed as resulting from a continued reliance on emotional schemes that had been formed earlier in one’s life.” [(64), p. 48].
3. *Bidirectional dyadic.* The explanation entails two characters, both of them are actively involved.
  Example: “Depression is indeed associated with <…> couples’ difficulty with solving problems, and less satisfaction (and dissolution) in a romantic or marital relationship” [(67), p. 41].
4. *Triadic*. The explanation involves three or more characters but only partially links them.
  Example: “Insecure parent–child attachment and parental conflicts have also been identified as early predictors of depression” [(67), p. 40].
5. *“Systemic” triadic.* The explanation involves three or more actors, linking them in a circular gestalt.
  Example: “FFT’s superiority over crisis management in the reduction of depressive symptoms was partially mediated by its effect on positive nonverbal interactions by family members” [(64), p. 50].

The content (causes) of each explanation was coded in five categories, three of which corresponded with those used by Schweizer et al. (69).

1. *Traumas and external events.* Mood disorders are attributed to events, which the client considers traumatic or constructs as external. Such events could also be positive, nonetheless, clients believe they have no control over them.
  Example: “Other events, such as occupational failure or job loss (sometimes called agentic stressors), have also been associated with depression from both theoretical and empirical sources” [(67), p. 41].
2. *Biomedical explanations.* Mood disorders are attributed to genetic or hereditary factors, to organic diseases, or to physiological dysfunctions of the client.
  Example: “In contrast, studies conducted in the afternoon found elevated cortisol levels in depressed individuals” [(67), p. 36].
3. *Personality traits.* Mood disorders are attributed to stable personality traits.
  Example: “The personality dimensions most germane to mood disorders are neuroticism and extraversion” [(64), p. 37].
4. *Intrapsychic conflicts.* Mood disorders are attributed to dilemmas or conflicts within the person.
  Example: “Beck’s model posits that depression is characterized by unrealistically negative views of one’s self, world, and future” [(64), p. 43].
5. *Interpersonal conflicts.* Mood disorders are attributed to interpersonal conflicts or difficulties.
  Example: “A substantial number of interpersonal factors have been associated with the onset, maintenance, and/or recurrence of depression across different phases of life” [(67), p. 40].

The first coder (EZ) identified and coded all explanations (*N* = 356) provided on mood disorders in both books (64, 67).

The second coder detected 73.1% (*n* = 88) of inference fields explanations and 50% (*n* = 61) of content explanations in the chapter Depression (67). For the inference fields, the inter-rater agreement is κ = 0.55, *p* < 0.0005, while for the content explanations Cohen’s kappa is higher (κ = 0.88, *p* < 0.0005).

The third coder analysed the chapter Mood disorders (64) identifying 19.4% (*n* = 45) of inference fields explanations and 19% (*n* = 44) of content explanations. The inter-rater agreement is from moderate, according to Altman (70) for the content explanations (κ = 0.51, *p* < 0.0005) to good for the inference fields (κ = 0.8, *p* < 0.0005).

### Data analysis

The first coder identified 356 explanations, on average 178 per chapter/text (range: 132–232). The data were analyzed by calculating the frequencies of units within the coding categories and by comparing differences between the two textbooks. The study design includes the following variables: inference field and content. In order to obtain an adequate frequency in each cell, the inference field variable was collapsed into its three main levels: monadic, dyadic, and triadic. Also, the content variable was clustered in three main categories: external causes (trauma and external events), intrapersonal characteristics (biomedical explanations, personality traits, intrapsychic conflicts), and interpersonal dilemma and conflicts.

### Natural language processing

To gain deeper insights into the content and visualize its predominant language patterns related to symptoms explanations, we adopted an alternative to human coding procedure and employed natural language processing (NLP) techniques using R (Version 2022.12.0 + 353) on the selected chapters (64, 67). Bi-grams (frequency > 4) were specifically selected due to their enhanced informativeness in capturing meaningful message associations compared to individual words (unigrams) (70).

For the purpose of our analyses, which focused on comprehending the overall trends within the two chapters, we combined them in this phase of the investigation. The objective behind employing NLP filtering techniques was not only to eliminate noise (72), but also to exclude irrelevant content, such as pairs of words that did not contribute to explanations of etiopathology, symptoms or therapeutic change (for example, words as “depression,” “disorder,” “patient” etc.).

## Results

### Examining the breadth of the inference field

**Figure 1:**
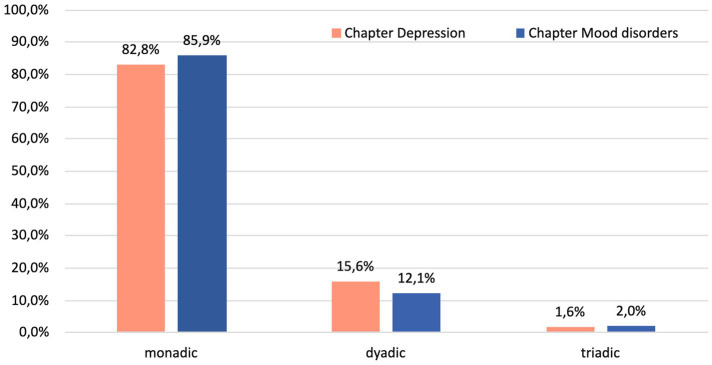
Percentage of monadic, dyadic, and triadic explanations of etiology and symptoms of mood disorders in two textbook chapters (*N* = 163).

With two expected cell counts less than five, Fisher’s exact test (2 × c) showed that there was no statistically significant difference between books in the distribution of explanations of symptoms and etiology between three levels (Figure 1), *p* = 0.84. The chi-square goodness-of-fit test indicated that levels of explanations were not equally distributed and handbooks resorted almost exclusively to monadic, with some presence of dyadic explanations (*χ*^2^(2) = 195.57, *p* < 0.0005).

**Figure 2:**
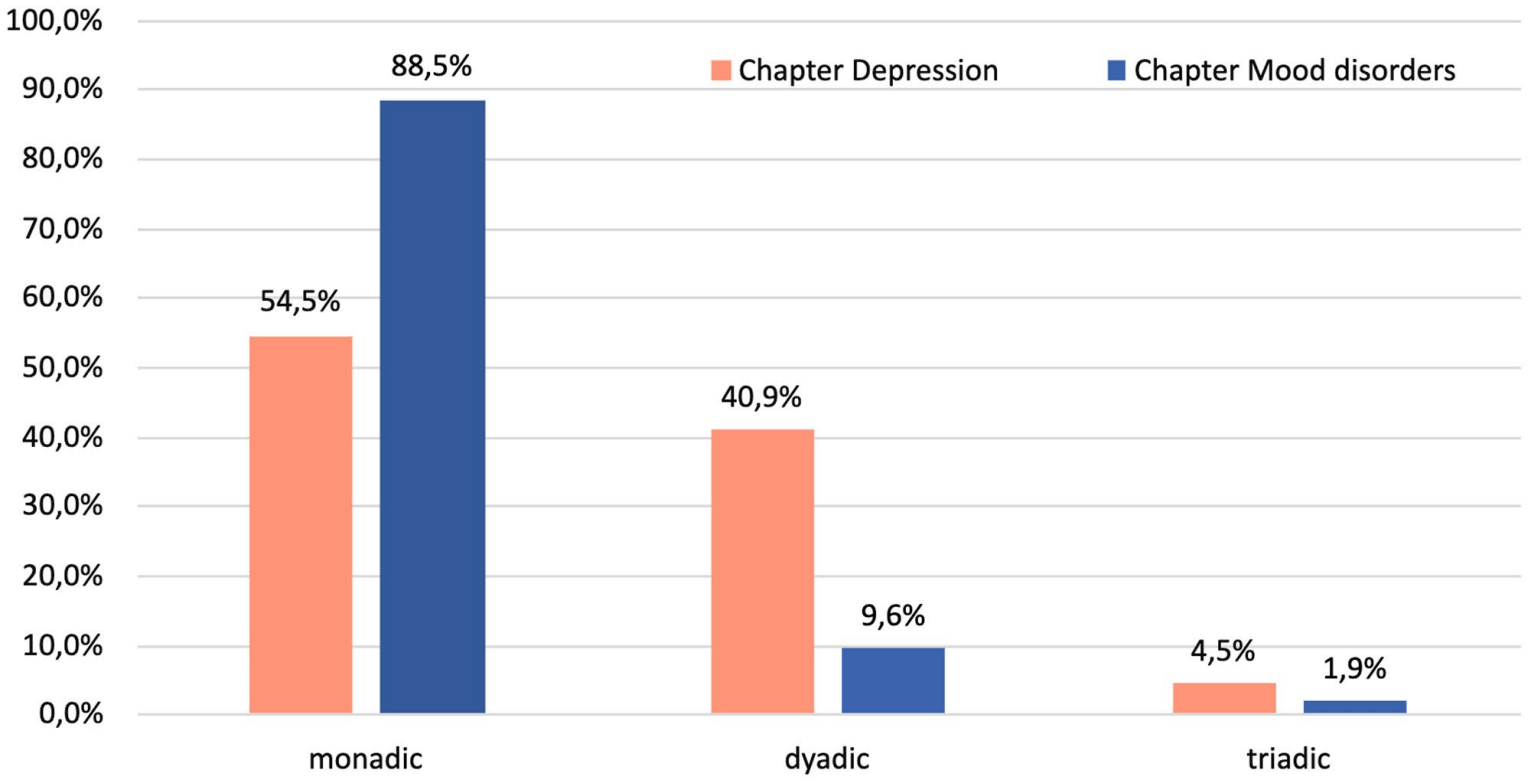
Percentage of monadic, dyadic, and triadic explanations of therapeutic change for mood disorders in the two textbooks (*N* = 148).

However, according to the results of Fisher’s exact test, there was a significant variation in the levels of inference between the book chapters (Figure 2) when it came to explaining therapeutic change (*p* < 0.0005). *Post hoc* analysis involving pairwise comparisons using multiple Fisher’s exact tests (2 × 2) with a Bonferroni correction showed that the difference was related to higher prevalence of monadic explanations and less dyadic explanations in the chapter “Mood disorders” (64), *p* < 0.000005.

The chi-square goodness-of-fit test indicated that levels of explanations of therapeutic change were not equally distributed (*χ*^2^(2) = 140.97, *p* < 0.0005).

### Comparative analysis of explanations across handbooks

There was no difference in explanations of etiology and symptoms between book chapters, as shown by a chi-square test of homogeneity (*p* = 0.596).

**Figure 3:**
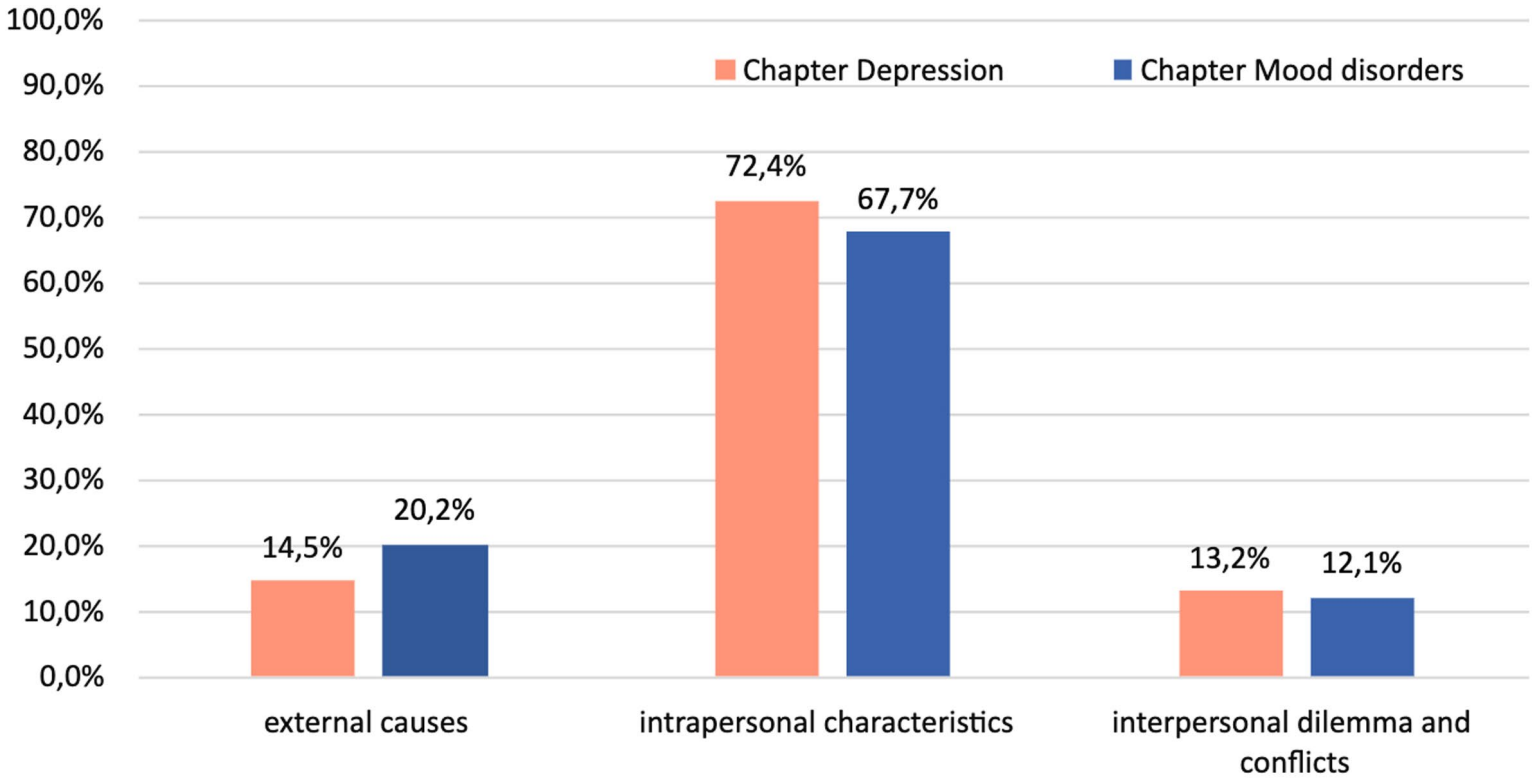
Distribution of explanations of etiology and symptoms of mood disorders across the two textbooks (*N* = 200).

The chi-square goodness-of-fit test unveiled a statistically significant imbalance in the distribution of etiopathogenetic explanations across categories in both textbooks (*p* < 0.005). Intrapersonal explanations prevail, as depicted in Figure 3, confirming our hypothesis (H2).

To check if biomedical explanations (H3) dominate in both texts, the content variable was clustered in four main categories: external causes (trauma and external events), biomedical causes (intrapersonal biomedical explanations), personal attributes (personality traits, intrapsychic conflicts), and interpersonal dilemma and conflicts, so that intrapersonal characteristics were divided into two subtypes, bio and not-bio. A chi-square test of homogeneity did not find a statistically significant difference between the texts (*p* = 0.617), so we analyzed them as a whole.

**Figure 4:**
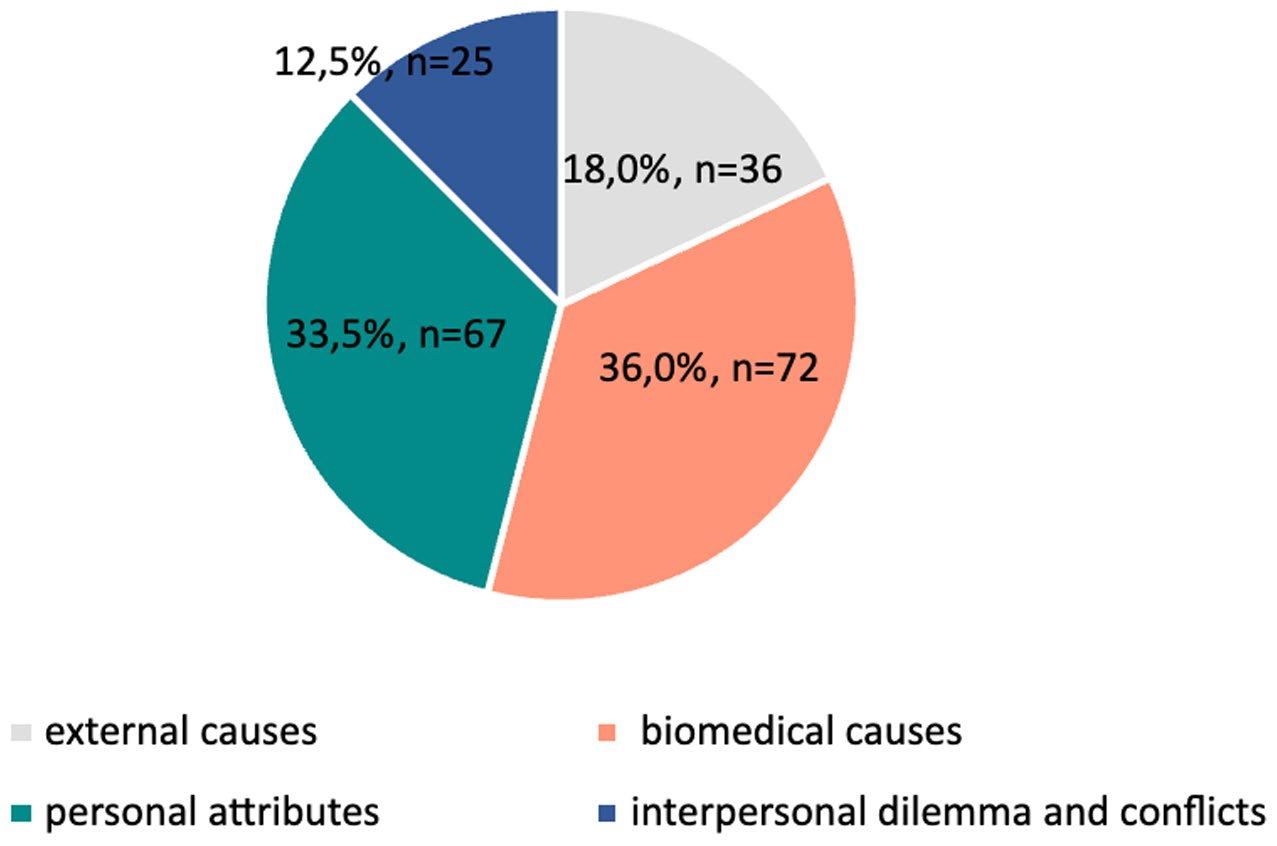
Percentage of explanations of etiology and symptoms of mood disorders in the whole corpus (*N* = 200).

Chi-square goodness-of-fit test confirmed unequal distribution of etiopathogenetic explanations in the corpus, *p* < 0.005. The main etiopathogenetic explanations for etiology and symptoms (Figure 4) of mood disorders were biomedical causes (36%), followed by personal attributes (33.5%). External causes were used in only 18% of cases, and followed by interpersonal explanations with 12.5%, hence fully confirming the H3.

### The general picture from a NLP perspective

**Figure 5:**
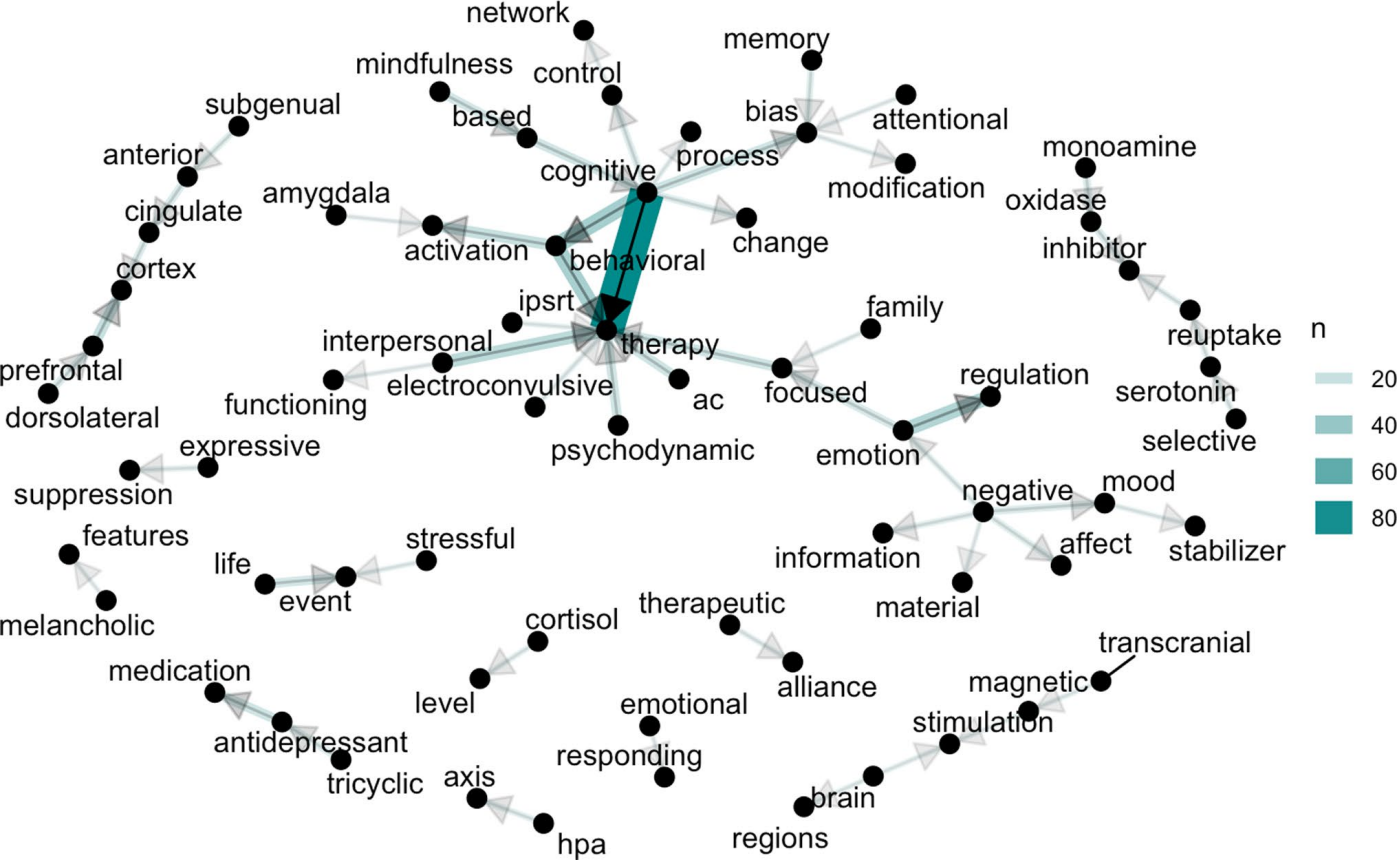
Bigram graph of content words related to explanations of etiopathology and therapeutic change of mood disorders. *AC therapy, Acceptance Commitment Therapy; HPA axis, hypothalamus–pituitary–adrenal axis.

The analysis of bi-grams (Figure 5) focused on explanations of etiopathology and therapeutic interventions revealed a notable disparity in their representation. There was a significant overrepresentation of cognitive therapy compared to other therapeutic approaches (with a total of 116 bi-grams), while interpersonal therapy emerged as the second most frequently mentioned, with only 25 pairs identified, and family focused therapy was mentioned only 6 times. Consistent with previous analyses, the application of NLP techniques also unveiled a noteworthy prevalence of language patterns pertaining to individual-based biological (for example, “monoamine oxidase inhibitor,” *n* = 11, cortisol level, *n* = 7 etc.) and other intrapersonal explanations (for example, “emotion regulation,” n = 29, “behavioral activation,” *n* = 20, etc.) (Table 1).

**Table 1:** Bigram pairs of content words associated with explanations of etiopathology and therapeutic change in mood disorders.

Bi-gram pair	*n*	Bi-gram pair	*n*	Bi-gram pair	*n*
Cognitive therapy	83	Monoamine oxidase	11	Bias modification	6
Cognitive behavioral	34	Negative mood	11	Brain regions	6
Behavioral therapy	33	Oxidase inhibitor	11	Brain stimulation	6
Emotion regulation	29	Tricyclic antidepressant	11	Cognitive process	6
Interpersonal therapy	25	Cognitive change	10	Family focused	6
Behavioral activation	20	Cognitive control	10	IPSRT therapy	6
Prefrontal cortex	20	Memory bias	9	Magnetic stimulation	6
AC therapy	17	Negative affect	9	Mood stabilizer	6
Antidepressant medication	17	Subgenual anterior	9	Stressful event	6
Cognitive bias	17	HPA axis	8	Transcranial magnetic	6
Focused therapy	17	negative emotion	8	Amygdala activation	5
Based cognitive	16	negative information	8	Attentional bias	5
Psychodynamic therapy	16	Reuptake inhibitor	8	Control network	5
Life event	15	Selective serotonin	8	Emotional responding	5
Mindfulness based	15	Serotonin reuptake	8	Interpersonal functioning	5
Emotion focused	13	Cortisol level	7	melancholic features	5
Anterior cingulate	12	Electroconvulsive therapy	7	Negative material	5
Dorsolateral prefrontal	12	Expressive suppression	7		
Cingulate cortex	11	Therapeutic alliance	7		

## Discussion

Our hypotheses were fully confirmed across both textbooks’ chapters examined, as they showed very similar explicative patterns. These findings support previous literature on similar topics but conducted with different methodologies (32, 49, 73).

In this paper we tackle the teaching and disseminating side of the issue, by considering how psychopathology both as a content and as a science or *discourse* is produced in handbooks that are adopted as academic textbooks in training courses for mental health professionals. We can consider handbooks the pillars underpinning and informing the approach to psychopathology research and practice undertaken by the new generations of freshly trained professionals.

As predicted, these two chapters are dominated by the stark prevalence of monadic explanations (up to 86%) and a very limited presence of dyadic explanations. Overall, the role of triadic or systemic explanations was negligible. This is in contrast with previous trends reported by Davies (1) and studies (46, 50, 51) on lay populations less exposed to the “urban legend.”

However, when it came to explaining therapeutic change, there was a significant difference in the breadth of inference fields between the two book chapters: the DeRubeis et al.’s (64) chapter “Mood disorders” mostly focuses on monadic fields and gives very little attention to broader explicative fields.

No less interesting is the etiopathogenetic content of the explanations provided by these handbooks. For years now, the scientific debate has tended to put etiopathogenesis between brackets. Indeed, we found no difference in explanations of etiology and symptoms between the two book chapters: etiopathogenetic explanations were dominated by intrapersonal explanations, with very limited space devoted to external or traumatic factors and even less to relational ones.

These unbalanced views of etiology and treatment factors can have important implications for the therapeutic indication, prognostic optimism and hope for recovery, as well as motivation and commitment to the therapy process. Several studies have demonstrated that participants exposed to biomedical explanations of depression tend to prefer pharmacological treatment, less likely express a willingness to seek psychotherapy, and are more inclined to believe that the solution of the problem lies beyond an individual’s control (74–77). Biochemical explanations in patients are associated with prognostic pessimism and less hope for recovery (74, 77–79), as well as increased stigma (80, 81).

Another pitfall in effectively helping people with depression arises from the tendency of both mental health clinicians and patients to perceive psychotherapy as less effective when provided with a biochemical explanation of a patient’s symptoms (75, 82, 83). This bias raises substantial concerns because, in such cases, a biochemical attribution can easily lead to overlooking the exploration of broader psychosocial and environmental factors. Consequently, this bias can contribute to a self-fulfilling prophecy, fostering the belief that psychotherapy is ineffective and, in reality, hindering effective coping with depression.

Given all that has been mentioned, we can underscore the risk that already vulnerable or oppressed groups, whose onset of depression might be related to poor social conditions, lack of resources, discrimination and power imbalances, may be further medicalized, disempowered, and marginalized.

The majority of explanations mention intrapersonal factors for disorders (either biomedical or those based on intrapsychic traits and conflicts), which are predominant also in other studies. The prevalence of intrapersonal biomedical explanations (mostly referring to the chemical imbalance theory and genetic factors) implies that these “urban legends” are resistant to change and indeed professional textbooks tend to provide unbalanced perspective of etiopathogenic processes with very little consideration of psycho-social factors and life events.

We argue that professional bodies, editors and authors should carefully examine what kind of explanations implicitly or explicitly are privileged in their textbooks and chapters. They should detail openly the ontological and epistemological underpinnings of their literature review and selection, and make evident their allegiances, as well as alternative and contrasting positions.

Our chances to better tackle mood (mental) disorders, and to make the best possible use of available care opportunities, could be significantly improved by measuring the extent to which the professionals’ views on depression and their care practices are in contrast with the current state of the art and with what is perceived as meaningful and relevant by the general and clinical population. We agree with Davis (1), that the biological “explanation” forecloses our engagement with meaning making, causing a hermeneutic closure.

## Limitations and future research

The exploratory nature of this study is also set by its main limitations. First of all, since the coding procedure is very thorough and time consuming, we analysed only 2 textbook chapters on mood disorders. Although consistent with previous studies (32), our findings may or may not apply to other textbooks and hence cannot be generalized. Some limitations are associated with the fact that both texts adopt the definition of depression according to the Diagnostic and Statistical Manual of Mental Disorders (13); consequently, differences may arise if other manuals are employed. Future investigations should increase the number of disorders and/or textbooks included.

Another limitation of the study could be related to the moderate inter-rater agreement for some parts of the coding procedure; this could due to the very complex and detailed analysis required and could be enhanced by more extensive and in-depth training.

There is not enough research on how clinicians’ awareness of their own mental state can influence the choice of intervention in favor of medication or protocolled treatments. In future research, it would be interesting to investigate, for example, whether the degree of implicit anxiety or the implicit sense of helplessness in medical professionals correlates with the recommendation to use antidepressants for their patients.

### A third way is possible: reconstructing psychopathology?

But is there an alternative to “over-diagnosis”? Can diagnosis mean something else? It did once. Even the two abovementioned UN and WHO groundbreaking reports seem to give for granted that there is no alternative diagnosis: we either go “diagnostic free” or label, pathologise and medicalise people in distress.

We would also stress that these models of mental health and recovery are imbued with hegemonic neoliberalism both in etiology and in healing, even more so for mood disorders: Emphasis on self-help, moral responsibility of individuals for their problems (42), emphasis on resourcing of individual strengths/capabilities ascribe everything to individual agency (brain chemicals), rather than institutional and social responsibility, structural inequalities (resources for welfare limited or undermined).

In this paper, we argue that another way is possible, if we move beyond nosographic and descriptive diagnosis only, and go back to the original attempts of hermeneutic psychopathology and diagnosis, which aimed at making sense of human distress and lived experience within an ongoing co-constructed conversation with people affected by it. Structural inequalities and discrimination, just like genes, cannot account alone for mental health etiology. Individual and relational meaning making processes and embodied positionings need to be encompassed to understand why anyone, no matter what their financial, privilege and genetic background, can struggle with psychopathology, and the other way around: why only one of two identical twins growing in the same ‘shared’ environment should.

If Cosgrove et al. (84) call for “an honest dose of gentle medicine” in psychiatry, we advocate for honest and meaningful psychiatry provision and training to start with.

An alternative framing, typical of systemic models, could involve understanding depression as having one or more important functions and signaling unmet needs, conveying a message to pay attention to some areas of life. Research shows that such an explanation of depression decreases stigma and increases the sense of autonomy and agency in overcoming depression in patients (85). There are different explanations for the role depression adjustment could play; for example, in certain circumstances, depression can be a functional way of relating with others and dealing with limitations (86), or saving energy in situations beyond control (87), or assisting in resolving relational dilemmas (3, 4, 88), etc. Perhaps the multiplicity of explanations is not a mistake but reflects the complexity and the need for exploration of the individual’s story and particular circumstances (meaningful relationships, and social forces at play) to uncover the meaning and function of symptoms. If we see the symptom as a creative adjustment to the contextual situation like systemic and gestalt (89) theories have theorised, it cannot be explained *a priori* and for all people with the same diagnoses.

## Data availability statement

Publicly available datasets were analyzed in this study. This data can be in the following two published books: (66, 67).

## Author contributions

LF: Conceptualization, Funding acquisition, Methodology, Supervision, Writing – original draft, Writing – review & editing. EZ: Data curation, Writing – original draft, Writing – review & editing. LG: Investigation, Supervision, Writing – original draft.
